# Finding their voices again: a media project offers a floor for vulnerable patients, clients and the socially deprived

**DOI:** 10.1007/s11019-013-9468-2

**Published:** 2013-02-13

**Authors:** Ralf Stutzki, Markus Weber, Stella Reiter-Theil

**Affiliations:** 1 Clinical Ethics, University Hospital Basel/Psychiatric Hospitals of the University Basel, IBMB, University of Basel, Schanzenstrasse 13, 4056 Basel, Switzerland; 2 Muskelzentrum/ALS Clinic, Kantonsspital St. Gallen, Switzerland

**Keywords:** Medical ethics, Media ethics, DU bist Radio, DBR, Patient participation, Vulnerable groups, Aidōs, Patient rights, Patient discrimination

## Abstract

‘DU bist Radio’ (DBR) is an award winning [DBR has been awarded with the “Catholic Media Award of the German Bishops Conference, Prädikat WERTvoll” (2011), the Suisse “Media Prize Aargau/Solothurn” (2010), the German “Alternative Media Award” (2009) and was nominated for the “Prix Europa” (2009)] monthly radio format that goes on air on three Swiss radio stations. The purpose of this program which was first broadcast in 2009 is the development of a new media format which—without applying any journalistic (or other) filter and influence—conveys authenticity of expression amongst society’s most vulnerable fellow citizens such as patients, clients and the socially deprived. So-called *marginal groups* are encouraged to speak for themselves, as a possible paradigm case for encouraging the inclusion of patients’ and relatives’ “unfiltered” voices in general and in clinical ethics as well. Before handing over the microphone to the groups in focus, a team of journalists, educated in medical ethics, over a period of 4 days, teaches them on-site radio skills and craft. Once this task is completed and the actual production of the broadcast begins, the media crew does not exert any influence whatsoever on the content of the 1-h program. Thus, the final product is solely created and accounted for by the media-inexperienced participants, leading to unforeseen and often surprising results. It is discussed that the DBR approach of fostering authenticity of expression can serve as an enhancement to today’s respect and autonomy oriented field of medical ethics.

## Introduction

There are many groups in our society, of which we know merely that they exist. Not only our lack of in-depth interest in them, but also their exclusion from societal resources which can be easily accessed by us at any time, deprive them of their right to fully live out their humanity. They have become stereotyped, categorized and even stigmatized minorities: “handicapped persons”, “patients”, “clients”, “detainees” etc. The media project “DU bist Radio” (*YOU are Radio*) aims at retrieving those groups out of their artificial social distance by providing them with a tool that allows them to articulate themselves freely, i.e. without applying any journalistic, methodological or ethical filter and influence thereby conveying authenticity of expression. These oftentimes so-called marginal groups^1^ are encouraged to speak for themselves as a possible paradigm case for stimulating the inclusion of patients’ and relatives’ “unfiltered” voices in the field of medical ethics as well. We claim a significance of this project for clinical ethics in the sense that it serves as an encouragement to dare involving patients and relatives more directly in ethical discussions; pioneer projects doing this have been reported rarely (Reiter-Theil 1998a), despite an overall societal trend to favor participation (Schicktanz 2009), which has become a prominent principle in the bioethics discourse (Reiter-Theil 2003; Borders et al. 2005; Weingart et al. 2011; Harun et al. 2012; Schicktanz et al. 2012). In clinical ethics support (CES) there is an ongoing discussion as to whether and how patients and relatives should and could have access to CES or be involved to speak for themselves; the topic developed from a completely neglected issue to an attractive debate (Reiter-Theil 2003; Newson et al. 2009). Besides the principled matter of unquestioned patient rights to access and transparency, there is also concern that not all experiences around ethical case discussion or consultation might be beneficial to those affected and destabilized by illness and suffering. Routine healthcare professional-patient conversation also deserves to be looked at in terms of roles and reciprocity, and even closely following the doctrine of informed consent in its predominantly intellectual meaning has been identified as being insufficient, e.g. in end-of-life situations (Reiter-Theil 1998b). However, in practical life, there are many areas where participation is less visible than discrimination or segregation; thus, we are more likely to err by offering too little rather than too much participation. One such area of neglect has certainly been the involvement of patients in the discussion of ethical issues in health care (Reiter-Theil 1998a; Frojd et al. 2011). And the question whether patients’ rights and participation ought to be extended to other fields of social life as well has yet to be raised. As far as active media involvement of patients is concerned, recent scientific discussion has focused primarily on the pros and cons of social media (Thielst 2011; Yamout et al. 2011; Glick and Yamout 2012; Sweet 2012), the role of the media in end-of-life decisions (Drake and Cox 2012) or the disclosure of celebrity patient information in the traditional media like newspaper, radio and television (Burkle and Cascino 2011). The issue of free media access for those who due to illness or other circumstances, which make them vulnerable in our society, are unable to participate so far has—to say the least—not been at the core of recent research.

Our paper will start with a detailed description of the ideas and the procedures behind DBR. This will be followed by a discussion of the theory this media project is built on, namely the assumption that the encounter with the “other” on an eye-to-eye level—with the forces being equally distributed—can only be achieved if those who are involved are guided by a concept that we want to call *aidōs.* Aidōs, according to Greek mythology a virtue required for men’s peaceful co-existence, is the road man must walk in order to approach the other in a manner that exceeds mere respect. This encounter, it will be argued, can only take place in an un-conditioned manner, i.e. without conditions (German: *un*-*bedingt*); meeting the other in this sense is a premise for all human self-understanding and self-realization.^2^

The experience and insights gained in the DBR media project so far will be analyzed along the following research questions: (1) To what extent does the application of the virtue of aidōs support our relation to as well as the condition of the vulnerable? (2) Can the DBR approach, which defines human encounter particularly as a transfer of competence and responsibility to the side of the vulnerable, encourage efforts to amplify the four principle based (Beauchamp and Childress 2001) medical ethics of our time?

## Background, concept and goals

### Starting point

The idea of developing this broadcasting format grew out of a longitudinal study that we conducted with Swiss amyotrophic lateral sclerosis (ALS) patients and their caregivers. ALS is an incurable progressive motor neuron disease, which in the later stage can lead to total paralysis. The average life expectancy of patients suffering from ALS ranges between 3 and 5 years from time of diagnosis. This study was authored by the Muskelzentrum/ALS clinic at the Kantonsspital St. Gallen and the Department of Medical and Health Ethics, Medical Faculty of the University of Basel^3^ and focused on end of life issues, suicidality, spirituality and quality of life of ALS-patients and their caregivers at an early and later stage of this fatal disease (Stutzki et al. 2012). In the process of conducting interviews^4^ that took place at the patients’ homes, a question arose for the responsible interviewer, who is also an experienced journalist,^5^ about the nature of the data being collected. On the one hand, it was clear that this research was based on current methodological standards of research and should provide valuable data for the ongoing ALS-research. On the other hand, the most private and intimate interview setting—i.e. patient homes—stimulated two unexpected insights and experiences which later on would provide the grounds for the theoretical foundation of the DBR media project:

- The interviewer specifically sensed a peculiar uncomfortableness due to the in-house setting. Asking the participants about most personal and existential matters (such as their attitude towards life prolonging measures, suicidality and the quality of the relationship between the patient and caregivers—usually wife or husband) in their private rooms to him felt like an almost forbidden and unacceptable intrusion into the lives of the participants, who undoubtedly had invited him to this proceeding. The interviewer decided to follow up on this experience, the results of which will be shown later in this article.
- The setting stimulated reactions and further statements of the participants, which the methodologies as such used in this research did not trigger.^6^ Upon completion of the study questionnaires and semi-structured interviews, when the recording device had been switched off and no further questions were directed at them, a majority of both patients and caregivers began to open up to the interviewer, thereby providing another quality of insights into their overall condition. This experience is not strange to social scientists (Devereux 1973) and we argue that this information, given upon completion of the interviews, provides a fruitful and valuable input for the on-going ALS-research as well as for medical ethics at large. In addition to the empirical research tools used in this study, patients and caregivers in a sense developed their own “methodology” that led to further insights into their coping strategies with regard to their disease. An example:

Upon completion of the interview, when the casual part of the meeting began, a male patient asked the interviewer to no longer address him as such: “Don’t call me ‘patient’. I have a name. I am a human being.” The interviewer immediately felt caught red-handed while a sentiment of shame (German: Scham, see Chap. ‘A methodology stimulated by mythology: the aidōs-approach’) arose within him. The study participant—most likely unintentionally—had unmasked the interviewer by mirroring an attitude that interpreted this relation as one where the powers had been distributed unequally—obviously to the disadvantage of the patient. A situation where the ‘I’ is the interviewer while the ‘You’ is the ‘patient’ serves as a convenient resort for the one who is asking the questions. It provides a distance between the two and categorizes those who are involved—possibly caused by the wish (or even need) for self-protection. Once, as happened in this case, these categories have been identified and resolved, the former I/You relationship turns into a common “We.” After all, the interviewer—just like the interviewee—has a first name and is a human being. The focus of this relation then switched towards that which was commonly shared. It unified and no longer separated.

The interviewee then invited the interviewer into another room in his house and began to share with him one of his life long passions: music. This room was filled with guitars, countless CDs and LPs. Due to the advanced stage of the ALS disease, the interviewee’s hands and arms were completely paralyzed. But he could still play music. With the help of a friend he had constructed a foot-guitar, which can be operated with toes. Without saying a word the “former patient” sat down and began to play.^7^

Experiences as this one were the starting point for developing DBR as a tool of expression for vulnerable groups. DBR calls them “Menschen mit einer besonderen Lebensgeschichte” (‘people with a remarkable story in life’).

### Background

“DU bist Radio” (DBR) is a public platform for those who are hardly recognized or even excluded by society. At the core of this media concept is free broadcasting time (120 min) which is being offered unconditionally and without any obligation whatsoever to the people in focus. The explorative and live character of this project—visiting and working with vulnerable groups on site (e.g. on the ward) makes DBR unique. These face-to-face encounters activate an interpersonal process that is an important feature of the concept. The DBR producers consist of a team of four professional journalists also educated in medical ethics, as well as a group of long-term unemployed persons striving for an occupational redeployment in the media field. Thus, not only a professional, but also a frail group of journalists works face-to-face with others who have to deal with severe life crisis or real life threats. Quarreling with these circumstances—as has been our experience so far and will be discussed—may also lead to a reassessment and even repositioning of one’s own allegedly difficult situation. Since DBR focuses particularly on people with a ‘special life-story’, i.e. vulnerable groups or individuals at risk, the approach towards them by all means must not be artificially created. The encounter has to take place at eye level, meaning that the other—despite his situation—is foremost seen in his humaneness and not in categories like e.g. ‘patient’, ‘addict’ or ‘disabled’. This can only be achieved if every member of the professional team involved in the DBR production knows about and accepts his own abysses: “If thou gaze for long into an abyss, the abyss gazes also into thee” (Nietzsche 1984, p. 82).^8^ In a certain sense this media concept requires a position of equality between the participants and the producers. This condition undoubtedly cannot be reached completely; nevertheless there exists no reason why a group of people—both ways—should not attempt to de-categorize^9^ each participant, emphasizing his humaneness only and aim at experiencing an “original position” behind a “veil of ignorance”, thereby assuming “that the parties do not know their conceptions of the good or their special psychological propensities. […] This assures that no one is advantaged or disadvantaged” (Rawls 1999, p. 11).

DBR is produced and has been broadcast since 2009 by the regional radio channel Kanal K in Aarau, Switzerland. Kanal K was founded in 1987 and is a non-commercial 24-h program with a keen focus on cultural contents. While a certain segment of broadcasting time is open to the public (“public radio”), the majority of the program is produced by two in-house editorial departments, which are directed by professional journalists. The first department is composed of up to 10 journalism-students who spend a 3 months traineeship (compulsory) as part of their academic program at the station. The second department “stage on air”, which produces DBR, consists of up to eight long-time unemployed persons who qualify for an occupational redeployment in the media field. They usually receive 6–9 months media training at the station. The Swiss Federal Office of Communication (OFCOM) and the federal unemployment insurance fund this training commitment of Kanal K. Kanal K and the co-broadcasting radio stations of DBR, Radio X in Basel and Radio RaBe in Bern, are members of UNIKOM, the Swiss organization of non-commercial radio broadcasters.

### Approach

For a period of 4 days a team of journalists visits the DBR groups on site (wards, therapeutic living communities, prisons etc.) and works with them towards preparing the upcoming production. A DBR group^10^ on average consists of 10–20 people. After a comprehensive introduction into the 4-day program the DBR participants split up into small working groups. It is important to point out that the DBR group members have been informed that they are not expected to define themselves in categories such as “handicapped” or “patients” during the course of the program. They are free to broach the issue of their suffering; they’re also free to choose a completely different horizon of content. Under the supportive yet non-directive guidance of the journalists these groups develop ideas and contents for their broadcast, which at the end of each day are put to discussion amongst all. As in good brainstorming, every idea is welcome and considered worth to be discussed. There is room for sharing dreams, talking about the present or past, about hopes and fears, and, of course, about what it means to live a categorized existence at the edge of society. Everything can, while nothing must be discussed. During the day each and every suggestion is put up to discussion amongst the whole DBR group which, as the production team takes on a role of non-intervention in this process as well, decides completely on its own about the themes that shall be presented in the program. The primary task of the production team during the 3 days of preparation for the recording is to assure that every participant has a chance to speak up and to put his/her ideas to discussion with the underlying rule that the input of each person is equally important and worth to be considered as everybody else’s. As soon as the ideas take shape and the group at large decides to include them in the program, the production team if necessary assists in the process of writing the script, particularly in light of the fact that “radio language” is a language of its own. Once the scripts are finalized, the DBR participants practice presenting them under the guidance of the journalists in front of a microphone. At the end of day 3, when all ideas and stories have been written down and presentation has been finalized, the DBR group—not the journalist—by majority vote decides on issues such as sequence or music selection and picks out those participants who will present the radio broadcast. Day 4 is the recording day. Depending on the size of the group and the number of inputs, the recording time for the final 2-h program is between six and 8 h.

**Fig. 1:**
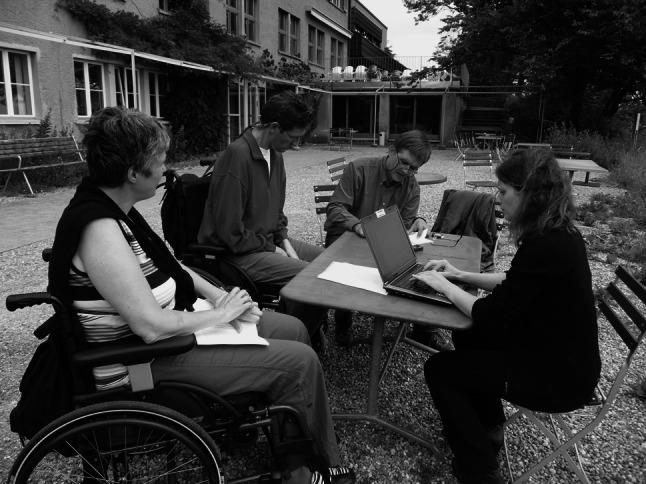
Gathering thoughts and typing the scripts: group work with people suffering from ALS

At the end of each production day the DBR journalists post a personal report about what happened during the day as well as photos and videos on social media such as the DBR Facebook page and the DBR YouTube channel,^11^ inviting the DBR participants (if they have access) as well as everyone interested in the production to comment and join the discussion online (Fig. 1).

### Project goals and their societal context

DBR aims at breaking down taboos and categories that deprive vulnerable groups of the chance to participation. The on-site approach enables the group in focus to take part in a media setting which up to then had been out of their reach. Free access to the media is a fundamental democratic right that the vast majority of society is able to execute. This right, however, for the most part cannot be practiced by severely ill patients, clients, inmates etc. provoking a question of uttermost importance: who decides about the allocation of rights in a democratic society—and on what grounds? As far as free access to the media is concerned, DBR assumes that there exists no justification for excluding others from their rights to participation. Furthermore, a society that withholds in particular its most vulnerable members the opportunity of free speech and independent presentation in the media adds to their deprivation of rights in general.

If for example people who are mentally challenged reclaim the medium for themselves, they send out a strong signal that in this area of social life there can exists equity between them and the supposedly “normal” and not disabled. A paradigm change becomes possible: the de-categorization signals the possibility to (re-) conquer areas of community life that until then had been inaccessible. And, of course, also the “other side”—media and society at large—can benefit since the democratization of the (here:) microphone can lead to new insights and contents. All of a sudden and to the advantage of all a discriminatory term and concept become unmasked and demystified: “handicap” (German: *Behinderung* 12) stands for obstacle (German: *Hindernis*). Unfiltered media access is a key to tear down those obstacles, which provide a fertile soil for socially convenient prejudices, fears and ignorance. Further, the transfer of journalistic expertise to these vulnerable groups is a strong sign that they—like everyone else—are both authorized *and* able to take on an equitable position among media professionals and to present their authentic issues and concerns to the public. This can serve as a model for other areas of life, in which equality, participation and integration of e.g. patients and disabled people have not yet been reached. An example:

Barbara, a 34 year old woman with trisomy 21, wanted to participate in a DBR production. When it was her turn to record, she sat down in front of the microphone and for 45 s merely moved her lips. Her caregiver explained that Barbara never speaks when she feels completely at ease. In such a situation she only moves her lips, articulating herself visually.

Forty-five seconds of silence are considered a “transmission hole” in daily radio business and must be—as is common broadcasting practice—avoided by all means. We decided, however, to broadcast Barbara’s contribution, since it was obvious that she had communicated with the listeners. As a matter of fact, Barbara’s contribution served as an advancement of the DBR concept: the problem of “not understanding” is—thanks to Barbara—now considered to be a problem on the side of the listeners. If they don’t understand, they will have to search for the cause.

### Stories told on DBR

The variety as well as concentration of contents, stories and thoughts expressed in the DBR productions so far is remarkable, as the following four examples shall show.

#### Children—setting their own agenda

The first DBR-production took place at “Kispex” in Zurich. “Kispex” is an institution offering palliative care for seriously ill or dying children. The production crew had prepared for a strenuous and intense time to be spent with a group of up to 14-year-old children suffering from a variety of (fatal) diseases. Before the production started it was clear to the journalists that the children would want to talk about issues related to their illnesses: e.g. about pain and fears; not being like other (healthy) children; the struggles of each day; the short lifetime left; the tense situation in their families etc. The production team had erred. The children did not spend a single word on issues related to their ill health. Instead, all of them talked about their future: Mario (11) brought along his keyboards, played some tunes and talked about his goal to become a professional musician. Marco (13) talked about his favorite subject at school (“vacation”) and presented the history of his favorite football team FC Zurich. His career goal was to become a sports reporter. Lara (14) discussed her plans to go on a safari 1 day. She liked predators and at night she dreamt about having a tiger baby on her lap. The children talked about the future—not about our expectations.^13^

#### How a fatal disease triggered a “wonderful” family experience

In another DBR-production André wanted to talk about his illness. André, 43, was suffering from devastating ALS. At the time we met him, he was sitting in a wheelchair and was unable to move his arms and hands. In our program he shared two most personal aspects of his life with the disease. One was about his 8 year old daughter and how she struggled to accept her father who had turned from a healthy man to a seriously ill person in a very short time. André from his childhood on had a great affection for handicrafts and had always hoped to pass this talent on to his child. Being together in the hobby room, unable to point towards anything, he now had to tell his daughter: “Watch where I am looking.” But whenever the point is reached where words do not suffice (e.g. when the daughter is not strong enough to saw a piece of wood) “all we can do is stop and do something else.”

André describes his disease as “sent by the devil”—and at the same time as the reason for a dream come true. For a long time his father had dreamt about a fishing trip to Norway—together with his children André, his twin-sister and younger brother. Father and siblings lived abroad. Despite André’s physical limitations they all got together and realized this long-cherished wish: “This was a wonderful vacation and we all got along great. I believe that without my illness this trip would never have been realized. All of a sudden everyone could spare the time and shared the desire to realize this dream.”^14^

#### I am Lilly—YOU are Lilly

We also produced in “Lilith” in Oberbuchsiten—a rehab center for addiction treatment for women and their children. Some of the participants chose to talk about their roads of the past, which led them into hard drug addiction. Almost all life stories were characterized by the experience of severe violence like rape in their childhood and youth. Even for experienced DBR-journalists it was difficult to listen to some of the stories told. And some of the women struggled as well when talking about their past.^15^ The “Lilith” women developed a remarkable acoustic tool, which made it at least a bit easier to tell their stories: together they created a kind of alter ego who they called “Lilly” and hence wrote their manuscripts in the third person, each and every one beginning her story with: “Lilly is …”

**Fig. 2:**
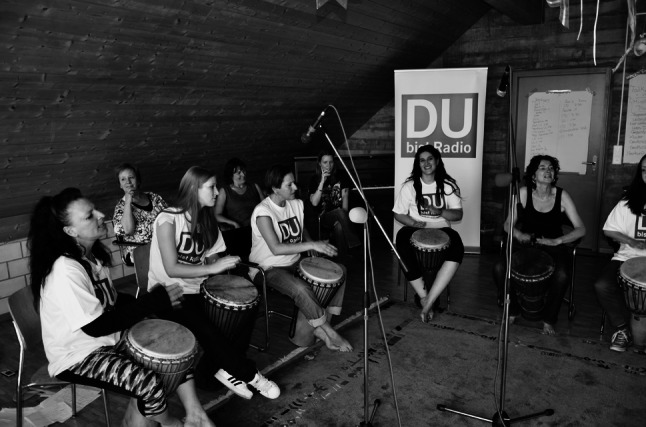
Recording a Djembe-Song: “Lilith”-women in Oberbuchsiten

At the end of these roundabout 1-min spots each story ended with: “YOU are Lilly”, echoed by the whispering choir of all the other participants: “I am Lilly, I am Lilly, I am Lilly …” This translation of transferring the own story into a third person, echoed by a whispering choir, undoubtedly has been one of the most magic moments in the DBR broadcast history so far (Fig. 2).^16^

#### I got myself a second face

Tobias lives and works in the “Stiftung für Behinderte” in Lenzburg along with 280 people with a variety of mental disorders. During 3 days of our production he was absolutely quiet and showed no sign of wanting to actively participate in the broadcast. During breaks we asked him how he was doing and if he wanted to contribute a story, a song or whatever to our production. He answered: “I’ll write something.” When we recorded DBR on day 4, Tobias came up to us during a break and pulled out a typed manuscript that he apparently had prepared on a computer. “I would like to record 2 pieces”, he said, and this is what he did. In piece 1 he described himself as a person with “Asperger’s Syndrome” (autism spectrum disorder) and presented a most detailed and profound analysis of this condition. Tobias’ second contribution was a summary of his childhood and youth, emphasizing the discrimination he experienced in school as well as during his apprenticeship: “I was always alone. I was never accepted in school—no matter what happened. And I was someone who did not defend himself. They said: ‘No, not him again. We don’t want him.’ And I started to change. I got myself a second face.”^17^

Tobias—along with other DBR participants in Lenzburg—taught us one important lesson: people with special mental conditions (whatever they may be) know when and how we discriminate them.

## A methodology stimulated by mythology: the aidōs-approach

DBR is an attempt to transfer an equal share of responsibility and power to the side of the vulnerable “other”. For a methodological orientation we adopted the aidōs-approach, which is a highly complex term found in ancient Greek mythology, particularly in the writings of Plato who even considered it an imperative for mankind’s survival. As it is the case with other concepts carrying emotional as well as moral connotations (Hollwich and Reiter-Theil 2012), aidōs has been characterized as both an “emotion-word” (Cairns 1993, pp. 7–13) and as a virtue (Kullmann 1998; Hogrebe 2004), the latter of which we choose to use in our analysis, leaning on Erffa that aidōs is “eine eigene Kraft, für die uns das Wort fehlt”—a power in itself for which we lack the terminology (Erffa 1937). Before we will sketch the mythological background of aidōs, we will describe the “methodological” aspects of the approach.

**Fig. 3:**
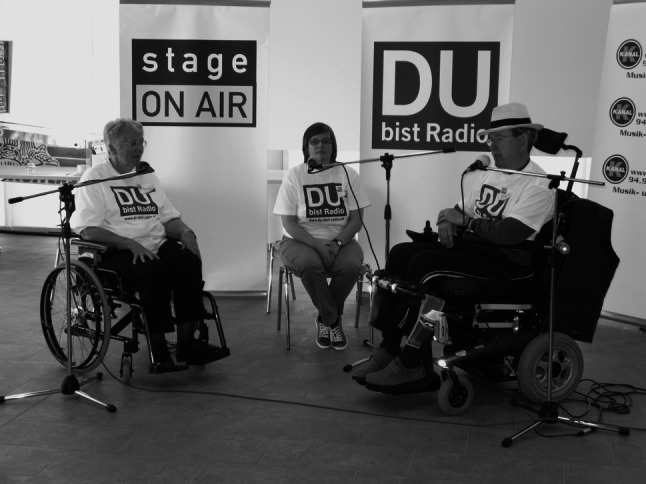
A talk about what it means to live with multiple sclerosis. Swiss Multiple Sclerosis Society, Zurich

Since its beginnings in 2009 DBR has been—and will continue to be—an evolving project. Both the varieties of the hundreds of people involved so far as well as their individual and unique inputs into each program have challenged the producers to continually react and adjust a concept that had started out as a ‘simple’ broadcast idea. Accordingly, it would be presumptuous on the authors’ side to claim that “DBR” rests on a clear cut methodology carved in stone. It simply cannot. Nevertheless, as this media concept continually advances, so does its theoretical framework: this ‘methodology in progress’ serves as a bridge connecting and interpreting our work with the vulnerable on the one side with the aidōs-approach on the other side. Aidōs, as will be shown, is a virtuous tool provided to all men particularly to guide them in their quest for knowledge. Respect, curiosity and tolerance, attitudes as important they may be, do not suffice in this endeavor if our *quest to find out* touches or even digs into those spheres that solely belong to our counterpart. Serving as a moral and emotional compass, aidōs requires the strict abidance to rules and principles: (1) The DBR-encounter has to take place within a framework where all parties involved hold a position of equality.^18^ (2) Structures of authority must be minimized to the smallest degree possible, the organization and responsibility of which lies in the hand of the majority (i.e. *participants* of the DBR-project). (3) Since in this media project the vulnerability of the participants not only becomes apparent physically (wards; cells; condition of patients), but oftentimes also through the most intimate stories shared during the production days, the members of the professional crew must for the sake of reciprocity be willing—whenever asked by the participants—to allow insight into their own vulnerability as well (cf. discussion on “abyss”, Chap. ‘Background’). Abiding by these three principles allows an atmosphere of trust and security to unfold. It is this approach that creates a unique setting where the story of the “other” all of a sudden can become “my” story (Fig. 3).

## Terminological clarification in the mythological context

Since the scientific discussion of the Greek term aidōs so far has not reached a widespread scholarly attention—let aside in particular Douglas L. Cairns’ remarkable work (Cairns 1993)—we will now have to analyze the terminology of adiōs-translations in order to grasp the rich meaning of the concept behind the word. The standard Greek-English lexicon (Liddell et al. 1996) translates aidōs as “reverence”, “shame”, “awe” and “respect”. The Greek-German lexicon (Pape 2005) translates aidōs as “Ehrfurcht”, “sittliche Scheu”, “Hochachtung” and “Scham, Unrecht zu tun”. Particularly the latter term (*Scham*) is of interest here, since it describes an emotion that has no equivalent expression in English. The German language distinguishes between “Scham” and “Schande” (engl.: “shame”). “Scham”^19^ desribes an emotion that keeps a person from performing an (immoral or forbidden) act, whereas “Schande” is an externally as well as internally imposed feeling following the act. The English term “shame” refers to both: a desire to “disappear from view” or to the “comportment that would avoid the emotion (the obverse of shamelessness)” (Lansky 1996, p. 769). Riezler bridges this linguistic gap between “Scham” and “shame” by concluding: “Aidōs is the shame that derives from reverence” (Riezler 1943, p. 463). Thus aidōs exceeds the level of reverence and mere respect. It is a state of “being awestruck” (German: “Ergriffensein”) while at the same time “experiencing reticent awe in light of the revered object” (Fahlbusch 2003, p. 2500) thereby representing a quality of emotional behavior that ought to be—and can be—achieved. In this sense, aidōs is a call for moral action. It approaches the “other” in adoration and herein recognizes his divine attributes: encounter guided by aidōs implies a “receding awe and shame, which prohibit hurting the dignified or even to approach it in an untactful manner” (Bollnow 1988, p. 99). There is both silence and amazement in the presence of a divine that is not only concealed above us but also *vis*-*à*-*vis* and revealed within the human “other.”^20^ To Protestant theological ethicist H. R. Niebuhr aidōs

keeps its distance even as it draws near; it does not seek to absorb the other in the self or wants to be absorbed by it; it rejoices in the otherness of the other; it desires the beloved to be what he is and does not seek to refashion him into a replica of the self […]. In all such love there is an element of that “holy fear” which is […] deep respect for the otherness of the beloved and the profound unwillingness to violate his integrity (Niebuhr 1956, p. 56).

In this holy fear man is “not allowed to touch everything” for there are “holy experiences before which they must take off their shoes and keep away the unclean hand” (Nietzsche 1984, p. 189). Nietzsche laments—interestingly enough—the cultured classes’ “lack of shame, the easy insolence of eye and hand with which they touch, taste, and finger everything.”

It has been discussed that encounter guided by aidōs must take on a reticent approach, accepting the boundaries set by the otherness of the counterpart. Aidōs enables man to abstain from crossing those “red lines”, which only on the surface appear to be manifold and different in origin. They all share—as the following discussion of two ancient Greek myths will show—one common ground: the human quest for (forbidden) knowledge.

In Plato’s “Myth of Protagoras” (Plato 1960) things on earth got out of control due to a well intentioned mind which pursued an honorable goal by taking the wrong turn. The virtue of aidōs in the end prevented mankind’s impending destruction and led Plato to develop his widely acclaimed anthropological declaration that all men are equally talented and qualified to decide on issues of justice in the polis (Kirste 2002):

After having created the earth the gods ordered Prometheus and Epimetheus to equip all mortal creatures with skills. Epimetheus was so eager to do the job that he asked Prometheus to step aside and merely serve as an inspector as soon as the distribution was completed. This was agreed, and so Epimetheus gave strength to the weak, armed some while he left others unarmed; he granted size to some in order to be able to protect themselves by their mere statue while he made others small and gave them wings in order to be able to escape. He shared all skills in such a manner that each mortal creature had the means to avoid extinction and to defend itself against all other races. Epimetheus even bestowed skills that protected all against the seasons: he clothed some with warming hair and others with thick skins that defended them against the summer heat and cold winters. He also provided a diverse food chain, including herbs of the soil for some, fruits of the trees for others, and to some he gave other animals as food. Epimetheus beyond a doubt meant well and would have completed the divine assignment to the gods’ absolute satisfaction, had he not in the process of bestowing skills lost oversight and forgotten one species. This is where the problems began. When Prometheus inspected the distribution, he found man to be completely unprovided. While all other animals and creatures were well furnished, man was naked, even without shoes. Man had no bed and no arms of defence. The hour in which the gods would examine the work of Epimetheus and Prometheus drew near, prompting Prometheus to take a wrong turn: he stole fire and the mechanical arts (required for its handling)—knowledge the human kind was not supposed to have—from the gods and gave them to man. The gods, of course, found out that man now had a share of the divine attributes. But without the art of government, these divine attributes would inevitably lead to man’s dispersion and destruction:

Zeus feared that the entire race would be exterminated, and so he sent Hermes to them, bearing justice (diké) and reverence (adiōs) to be the ordering principles of cities and the bonds of friendship and conciliation. Hermes asked Zeus how he should impart justice and reverence among men: Should he distribute them as the arts are distributed, that is to say, to a favoured few only, one skilled individual having enough of medicine or of any other art for many unskilled ones? “Shall this be the manner in which I am to distribute justice and reverence among men, or shall I give them to all?” “To all,” said Zeus; “I should like them all to have a share; for cities cannot exist, if a few only share in the virtues, as in the arts. And further, make a law by my order, that he who has no part in reverence and justice shall be put to death, for he is a plague (Plato 1960, 320d–322d).

In Plato’s myth man shares in forbidden aspects of the Divine (here: knowledge and crafts) and, in consequence, needs the virtue of aidōs in order to secure his survival. And he is free to decide whether or not he wants his life to be guided by it: Zeus’ answer to Hermes implies the possibility that man might not want to have a share in it. It is a decision in favor or against a moral law man is able to give himself.”^21^

What happens when the balancing tool of aidōs is purposely neglected is shown in Sophocles’ play *Philoctetes*. Even though there is no mentioning of the word aidōs in the play, the text “none the less provides us with a perceptive and convincing representation of the emotion in circumstances in which the ethical suppositions on which it rests are put to the test. The question of aidōs is raised by the issue of deceit” (Cairns 1993, p. 250). This ancient myth illuminates particularly the aspect of *Scham* involved in the aidōs-concept.

A snake in the Trojan War had bitten the warrior Philoctetes. Because of his terrible agony and foul smelling wound Odysseus banned him along with his magic bow to the desert island Lemnos where he was left to live all by himself. Ten years later the seer Helenus foretells that the Greeks can only conquer Troja if they possess Philoctetes’ magic bow. So Odysseus sails back to Lemnos where he asks his servant Neoptolemos to trick Philoctetes into handing out the bow (which in this story stands for knowledge the Greek were prohibited to gain): “I know, son, that by nature you are unsuited to tell such lies and work such evil. But the prize of victory is a sweet thing to have. Go through with it. The end justifies the means, they’ll say. For a few short, shameless hours, yield to me. From then on you’ll be hailed as the most virtuous of men” (Sophocles 1986). Neoptolemos was torn between right and wrong: “I do not want to make things hard for you. But I far prefer failure, if it is honest, to victory earned by treachery.” Odysseus stuck to his guns, trying to “forestall [Neoptolemos’] aidōs” (Cairns 1993, p. 251) by conjuring him that this one lie would lead to salvation. Neoptolemos gave in, not without asking his master a most crucial question, foreseeing, that by tricking Philoctetes something essential inside of him would have to fall out of equilibrium: “How could one say such things and keep a straight face? […] Then let it be so. I will do what you order, putting aside my sense of shame.” Later on, when Philoctetes finds out about the deceit, he charges Neoptolemos for having given up a part of his essential human nature: “How you have betrayed me! Are you not ashamed to look at me, who have kneeled to you, the suppliant, you bitter ones?” […] This is atrocious! He’s not speaking to me. He won’t even look me in the eye, as if he’ll never give me back my bow.” A straight face and the ability to look someone in the eye is the highest expression of a humanity guided by aidōs. It is an unspoken yet universally understood language which guards the rules required for living together in peace and freedom. To abandon this “virtue mechanism”—like Neoptolemos did—means to abandon a fundamental anthropological standpoint by treating man merely as a means, not as an end (Kant 1978). By putting aside the sense of shame (“Scham” as a self-inflicted emotion which prohibits man to commit the immoral act) a person needs to give up a share of his humaneness: “A man has to knock down (“niederknüppeln”) something inside of him in order to be able to look someone straight in the eye while lying to him. […] For Neoptolemos, to abdicate shame is equal to abdicate one’s own nature” (Spaemann 2011, p. 217).^22^

## Results

**Fig. 4:**
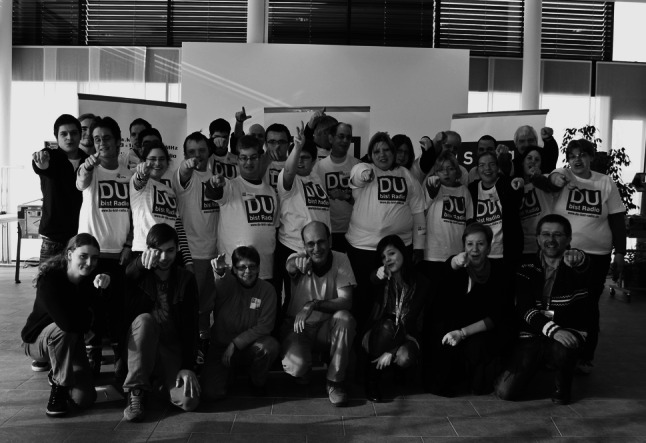
DBR group and production team celebrating at the end of the recording day. Stiftung für Behinderte in Lenzburg

Since 2009, close to 300 people have produced nearly 40 DBR broadcasts (Fig. 4). The productions have shown that the concept and approach work in the sense that collaboration and participation was obtained in all cases leading to significant results. The core of the DBR experience has been the oftentimes unforeseen and thus surprising content of each broadcast that could not have surfaced, had the state of the art journalistic tools and procedures been applied alone. Having been inspired by people participating in a “patient” study, DBR has shown that there exists another layer of truth^23^ behind the data structure that rigorous empirical research is incapable of finding. Rather, the aidōs-approach is capable to uncover this layer; it is possible that these truths belong to a sphere that should not be entered.^24^ The mythological and religious texts cited in this article, however, have shown that man in his eternal strive for knowledge is notoriously crossing those borders he is not supposed to pass—and therefore needs guidance by virtue.

By handing over its most important tool—the microphone—and by taking a backseat during the production process, the DBR producers also transfer the substantive sovereignty to the vulnerable groups at stake, thereby contributing to the disaggregation of the original role distribution (e.g. ‘journalist’ and ‘patient’). Numerous feedbacks and responses^25^ written to the DBR team at the end of the production show that the aidōs-approach indeed launches a process of development for each participating individual and the group as well: the “self-confidence of the participants has increased”; groups “have been blessed”, are “thrilled” and the “the community feeling has improved”; DBR is a “unique experience for the community” etc.

Even the production team benefits from this broadcast approach. Particularly members of the professional team have entered into a process of shifting the focus of their self understanding from *being* to *becoming*; other members of the DBR team, who have been long time unemployed and participate in the production as journalistic trainees, talk about their need to reassess their own situation: encountering vulnerability apparently has taken place on both “sides”, offering the chance for re-evaluation and repositioning of one’s own situation of life.

Furthermore, the DBR broadcasts create new listening habits and at times challenge the audience: Barbara’s contribution of 45 s of silence^26^ for example scrutinizes our commonly shared idea of what “good” radio should sound like and questions the status quo. A transmission hole, instead of leading to irritation alone, can turn into a silence that is worth to be borne. DBR participants reinvent the sound, content and format of broadcasting over and over again—without any input given by journalists and not a single question asked.

## Discussion

“DBR” started out as a project aiming at giving those, who oftentimes are considered to live at the edge of society, their *voices* back, thereby also reinstalling fundamental democratic rights they have been deprived of. It has been and still is the position of the DBR authors that granting a right not always comes along with the actual possibility to exercise it. With this position in mind the DBR experience would like to stimulate a discussion about the role and position of the vulnerable in the current medical ethics debate and about the conclusions we might draw from there for health professional-patient conversation. We hold that principle-based medical ethics, unquestionably important as it is, will not reach the fullness of its potential if it is applied only to those realms of life, which define the vulnerability (e.g. illness) of a person. Even the most well intended concept of (e.g. patient) rights may unintentionally run danger of discriminating instead of supporting the group at focus if the rights offered also promote the group’s categorization. Autonomy, justice, beneficence and non-maleficence, for example, must—as guaranteed rights and principles—also reach all other areas of man’s existence; areas such as passions, hopes, dreams, and individual circumstances which all add to the fullness of his humaneness. We believe that addressing the wholeness of the individual can also be a fruitful approach in the patient/physician setting: if, e.g. a physician invites a seriously ill patient to participate in the process of choosing one of the available treatment options, the outcome generated should also be evaluated in light of the question whether or not the role distribution of the two involved has been upheld or whether sovereignty in all its radicalness has been shared into equal parts. Furthermore, patient rights exercised in the sense that a certain decision has been made upon having given informed consent are at least questionable if the person finds himself thereafter alone and again in the ‘patient’s corner’. While the development of patient rights with its keen focus on autonomy and respect over the past decades beyond a doubt has led to an eminent improvement of the overall situation of society’s most vulnerable groups, we believe it is indispensable to take one further step: the *non*-vulnerable side (which includes the majority of us and comes down to e.g. caregivers and alike) must get even more actively involved into the process of dealing with those who are in need. The aidōs approach can be a fruitful tool in helping us not only to grant and maintain respect, but also to share and transfer responsibility.

While in the beginning days of the production the theoretical framework had by all means not clearly been developed, it soon became clear to the members of the journalist team that an attitude of mere respect towards the target group would not suffice and that they had entered a sphere in which they were *not allowed to touch everything* and where they had *to take off their shoes*.^27^ Studying the philosophical aidōs-literature on human encounter was the key to understanding the DBR experience. Man can only fully experience his own humaneness and grasp his identity with the help of the other. Becoming is only possible by entering into a relation that accepts the boundaries set by the other. And it is aidōs, this almost forgotten virtue, freely distributed by the ancient gods to all men, that enables man to enter into this relationship.

The DBR experience so far has shown that the aidōs-approach once applied can enhance the overall situation of those who due to their weakness have been categorized and thereby experience discrimination. By ‘acoustically stepping out’ of their wards and institutions, the DBR participants also step out of an identity that has never been their own in the first place: it had been imposed from the outside. This courageous move into the public view may lead to a as we believe long overdue and desperately needed discussion, at the core of which lies the question whether society needs to re-asses the role, value and potential of its most vulnerable groups.

## Footnotes

1 DBR has produced programs with groups of people suffering from e.g. amyotrophic lateral sclerosis (ALS), multiple sclerosis (MS), eating disorders; children in a hospice; people with psychiatric disorders, clients in rehab centers for addiction treatment, people in facilities for the handicapped, inmates in maximum security prisons.

2 This approach rests on the dialogical school of philosophical thought developed by thinkers such as Martin Buber: “I become through my relation to the Thou” (Buber 2005).

3 See: http://klinische-ethik.unispital-basel.ch.

4 The questioning consisted of validated questionnaires, rating scales and semi-structured open and tape recorded interviews.

5 First author.

6 Especially in empirical research settings with patients, questions and questionnaires oftentimes emphasize the interviewer’s quest for knowledge (which includes his/her attitude, presuppositions and prejudices) rather than encouraging the intervierwee’s spontaneous expression (Reiter-Theil 2012). Systematic methodologies like the Grounded Theory (Glaser and Strauss 2005) acknowledged this dilemma and made a great contribution to bridging this gap between theory and empirical research; nevertheless, the problem of an artificial setting where the interviewer faces the interviewee has not been eliminated.

7 Video: http://www.youtube.com/watch?v=U7XkpHaTwps.

8 In 1984 the first author met Pastor Dr. Werner Koch at his home in The Netherlands who shared with him a remarkable encounter. Koch had been a student and member of the Confessing Church in Nazi-Germany, which led to his confinement in the concentration camp Sachsenhausen. After the end of WW II, Koch picked up his theological studies, enrolling in a lecture given by Protestant theologian Karl Barth whom Koch greatly admired. At the end of class Koch approached Barth who—still in the lecture hall—had lit his pipe and was getting ready to leave. Upon introducing himself, Koch extensively confessed his adoration for the renowned dogmatist Karl Barth who, puffing on his pipe, answered with but one sentence: “I know my abysses, Mr. Koch.”

9 This approach of overturning hierarchies has been advocated by the semiotic analysis of “deconstruction” (Derrida et al. 1981).

10 Groups may apply for participation in this project.

11
http://www.facebook.com/dubistradio, http://www.youtube.com/user/dubistradio, http://www.dubistradio.ch.

12 This word in the German language still is also used for describing obstacles that prevent the “normal” to move on: e.g. *Verkehrsbehinderung* (obstruction of traffic).

13 Names used with permission.

14 André’s stories can be listened to online: http://youtu.be/mdv_PiKT9B0.

15 In order to avoid e.g. re-traumatisation and to assure the wellbeing of the DBR participants at all times, DBR works closely together with the institutions’ therapeutic and medical staff during the production.

16 Three Lilly-Spots can be listened to online: Spot 1 http://www.youtube.com/watch?v=heUi2f6xHjU&feature=plcp, Spot 2 http://www.youtube.com/watch?v=M5stOj3fojM&feature=plcp, Spot 3 http://www.youtube.com/watch?v=GaWpFOH0uDQ&feature=plcp.

17 Tobias’ contribution: http://www.youtube.com/watch?v=rrNydAiNhwo&feature=plcp.

18 To underline this, on day 1—after an introduction into the project—the DBR producer officially opens the “editorial meeting” and henceforth addresses the participants as “colleagues.” DBR has received numerous feedbacks documenting that this position of equality can be realized: “You made an editorial meeting out of the ‘*Harmonie*’ [institution for long-term drug addicts in Langenbruck]. There was no difference between the residents and the radio producers. Very impressive and sensitive. It is a privilege to be working with you.” (Jürg Lützelschwab, director *Haus Harmonie*, January 15, 2013. http://www.facebook.com/dubistradio).

19
*Scham* covers manifold emotional aspects: it also refers to the malaise of a person with his/her nakedness. To ‚cover your private parts’ in German translates as “die Scham bedecken.” The feeling of *Scham* causes a person to blush. See also FN 22.

20 Albert Schweitzer even urges the extension of aidōs to *all* life (Schweitzer 2003).

21 Was kann denn wohl die Freiheit des Willens sonst sein, als Autonomie, […] sich selbst ein Gesetz zu sein?” (Kant 1978).

22 The emotion of shame in conjunction with the human strives for forbidden knowledge also plays a significant role in religious writings, particularly in the scriptures of the monotheistic religions. In the history of creation the Judaic-Christian and Muslim texts report on how man—despite the divine orders—ate the fruit from the forbidden tree of knowledge. Prior to this act man was not aware nor ashamed of his nakedness (Genesis 2:25). When they had tasted of the tree, their shame became manifest to them (Quraan, Surah 7:22 and Genesis 3:7).

23 For an in-depth discussion of definitions, criteria and theories of truths see also Kirkham (Kirkham 1995).

24 cf. e.g. “Lilly”-stories, Chap. ‘I am Lilly - YOU are Lilly’; also: “(forbidden) knowledge”, Chap. ‘A methodology stimulated by mythology: the aidōs-approach’.

25 Feedback to the DBR productions has been published online: http://www.du-bist-radio.de/html/dbr_feedback.html.

26 See Chap. ‘Project goals and their societal context'.

27 cf. Nietzsche citation, Chap. ‘A methodology stimulated by mythology: the aidōs-approach’.
